# Real-time detection of Wi-Fi attacks using hybrid deep learning models on NodeMCU

**DOI:** 10.1038/s41598-025-18947-2

**Published:** 2025-09-15

**Authors:** Mohamed Hussien Moharam, Kareem Ashraf, Hussien Alaa, Mostafa Ahmed, Hesham A. El-Hakim

**Affiliations:** Department of Electronics and Communications Engineering, Misr University for Science and Technology, P.O. Box 23546, Sixth of October, Giza Egypt

**Keywords:** Wi-Fi monitoring, NodeMCU, RSSI, Deauthentication attacks, Hybrid AI models, LSTM_LR, GRU_LR, RNN_LR, Network anomaly detection, Real-time security, OLED display, Temporal pattern recognition, Edge computing, Machine learning, Deep learning, Energy science and technology, Engineering, Mathematics and computing

## Abstract

This paper presents a real-time, lightweight system for detecting Wi-Fi deauthentication (DA) attacks that uses the NodeMCU ESP8266 microcontroller for live packet sniffing and feature extraction. Tailored for low-power IoT environments, the system combines the sequential learning capabilities of Long short-term memory (LSTM), Gate recurrent unit (GRU), and Recurrent neural network (RNN) with the interpretability of logistic regression (LR). These hybrid models analyze Wi-Fi traffic in real time to detect anomalous behavior based on key metrics such as Received Signal Strength indicator (RSSI), DA, packet count, and Signal noise ratio (SNR), which are also displayed live on an OLED screen. The proposed framework uniquely integrates hybrid temporal deep learning with interpretable classification through (LR) in an ultra-low-cost embedded data acquisition setup through NodeMCU, addressing a gap in existing intrusion detection research that often focuses on either cloud-based processing or non-interpretable model. The system was trained and validated on a dataset of over 5,600 labeled samples collected under varied network conditions. Among the evaluated models, GRU_LR achieved the highest accuracy (96%) and demonstrated superior performance in identifying minority-class threats. By combining explainable AI with cost-effective embedded sensing, this work delivers a practical and transparent intrusion detection approach that can be readily adapted to diverse IoT and wireless security contexts.

## Introduction

Wi-Fi networks are the backbone of today’s communication network and provide connectivity at homes, offices, and public establishments. With increasing dependency on wireless technology, it is also essential to make networks efficient, reliable, and secure. Despite fantastic technological developments, Wi-Fi networks remain prone to performance degradation and hostile attacks. Interference in signals, busy bands, and environmental interference are some of the challenges to network reliability. For example, the 2.4 GHz band, which is congested with consumer equipment, is plagued by poor non-overlapping channels, whereas the less congested 5 GHz band is plagued by lessened range^1^.

Signal-to-Noise Ratio (SNR) is among the most important Wi-Fi performance metrics, being the desired signal power to background interference noise power ratio. Smooth and good-quality connections are indicated by high SNR, whereas low SNR results in data loss, lowered throughput, and repeated disconnections. Physical and electronic interference due to domestic appliances, as well as proximity to access points, are contributing factors^2^.

With the progression of wireless technology, threats to it also progress. Existing lightweight IDS implementations mainly depend on shallow learning models or fixed thresholds, which limit their ability to capture complex temporal attack patterns^3^. In contrast, our work combines deep temporal sequence modeling (LSTM/GRU/RNN) with a lightweight classifier (Logistic Regression) on a resource-constrained edge platform. This hybrid design enables efficient real-time detection with reduced computational cost. Machine learning (ML) and artificial intelligence (AI) techniques offer dynamic solutions that can analyze a significant amount of network traffic to identify typical behavior and alert on atypical patterns. Regarding Wi-Fi security, ML models are most helpful in identifying behavioral outliers, such as sudden spikes in deauthentication packets, thereby reducing false positives and enhancing threat response^4^.

The most aggressive of these attacks is the deauthentication attack, which targets an 802.11 protocol vulnerability to abruptly disconnect clients from the air. It may be used for launching denial-of-service (DoS) attacks, permitting man-in-the-middle (MITM) attacks, or hijacking sensitive user credentials. Countermeasures for such vulnerabilities in real time, especially for low-energy or resource-limited applications, are critical^5^.

However, few existing solutions combine real-time anomaly detection with low-cost, edge-based deployment using interpretable models, a critical need in constrained environments such as IoT networks.

To address this challenge, this work presents a robust, real-time Wi-Fi security monitoring system utilizing the NodeMCU ESP8266 microcontroller for live packet sniffing and feature extraction, while model training and inference are performed on an external computer. The system monitors key wireless parameters, including packet count, RSSI, SNR, noise level, and the rate of deauthentication packets, and displays them on an OLED screen for live inspection. A significant strength of this work lies in the creation of a real-time, application-specific dataset, collected directly from the NodeMCU during live traffic conditions. Over 5,600 labeled samples were gathered under diverse network environments capturing authentic temporal fluctuations and realistic interference patterns which ensured that the training data reflected the dynamic nature of real-world scenarios.

To analyze the collected data, we employ hybrid deep learning models that combine the temporal learning strengths of LSTM, GRU, and RNN architectures with the interpretability and efficiency of logistic regression (LR). Traditional models often struggle to capture the sequential dependencies inherent in time-series Wi-Fi traffic or fail to provide interpretable outputs particularly in constrained edge environments. The hybrid approach addresses these limitations by offering both robust temporal pattern recognition and lightweight, explainable classification, making it highly suitable for real-time, embedded security applications. While reference^23^ demonstrated the effective use of deep learning models on the standardized UNSW-NB15 dataset, our work introduces several critical advancements that address gaps in real-world applicability and methodological depth. First, whereas^23^ utilized a generic benchmark dataset, we have constructed and validated our models on a novel, custom-built dataset integrating specific IoT-relevant features like RSSI and DA levels, which directly enables finer-grained multi-class attack detection in real-world Wi-Fi environments. Second, beyond mere accuracy, we place a strong emphasis on deployability and resource efficiency. While^23^ employed a substantial 200 epochs for training a resource-intensive process suitable for offline analysis—our hybrid architecture was meticulously optimized to achieve superior performance with significantly lower computational overhead, making it viable for real-time inference on resource-constrained hardware like the NodeMCU. Finally, our proposed hybrid ensemble methodology (LSTM/GRU/RNN integrated with LR) moves beyond the single-model paradigms to offer a unique combination of temporal feature extraction and interpretable classification, providing both high accuracy and actionable insights not delivered by the models in^23^. This focus on a specialized dataset, hardware efficiency, and an interpretable hybrid model constitutes a novel contribution aimed squarely at practical IoT security deployment.

The novelty of our work is fourfold: First, we introduce a novel hybrid ensemble architecture that cascades temporal feature extractors (LSTM, GRU, RNN) with a logistic regression classifier, uniquely balancing high accuracy with model interpretability. Second, we move beyond standardized benchmarks by constructing and utilizing a custom, multi-class dataset integrating RSSI and DA levels for fine-grained attack detection in real Wi-Fi environments. Third, we provide unprecedented resource usage benchmarks that prove the practical viability of our system for real-time deployment on constrained IoT hardware like the NodeMCU, a key advancement over pure academic approaches. Finally, we establish technical depth and statistical rigor through a comprehensive hyperparameter configuration, detailed per-class metrics (precision, recall, F1), and a rigorous comparative analysis against Random Forest baseline, conclusively demonstrating the superiority of our method.

The primary contributions to this work are shown below:

- The development of a deployable, real-time dataset, and cost-effective Wi-Fi anomaly detection system utilizing NodeMCU and OLED visualization.
- A hybrid artificial intelligence framework that integrates deep sequential models with logistic regression to enhance interpretability and classification performance.
- Demonstrated an accuracy rate exceeding 96% in detecting multi-level deauthentication attacks across diverse network conditions.

The paper maintains a coherent flow in its presentation of the system development and assessment of the proposed system. The section "Related work" discusses related work in Wi-Fi security, anomaly detection, and hybrid deep learning approaches, noting the gaps this research aims to address. The section "Methodology" shows the methodology, including data collection using the NodeMCU, feature extraction, and the construction of hybrid models based on LSTM, GRU, RNN, and LR. The section "Results and discussion" presents experimental results, comparing the models’ performance using all evaluation metrics. The section "Conclusion" concludes the paper with a discussion of the main findings and practical issues, and future research, including hardware upgrades, larger datasets, and more sophisticated modeling techniques to enhance real-time responsiveness.

## Related work

Machine learning has emerged as a promising solution for resolving security attacks in Wi-Fi networks, specifically for the detection of complex and dynamic attacks. Deep learning algorithms such as Long Short-Term Memory (LSTM) networks are widely adopted for their ability to capture nonlinear, time-varying patterns in sequential data. For instance, the work of^6^ introduces a number of LSTM enhancements, including optimized cell states and convolutional variants, that facilitate improved performance on sequential and multidimensional data tasks. The applicability of LSTM goes beyond fields such as sentiment analysis, denial-of-service (DoS) attack detection, and malware classification^7^.

Simplified Gated Recurrent Unit (GRU) variants such as GRU1 and GRU2, explored in^8^, offer a lightweight alternative to LSTM while maintaining performance on tasks like digit recognition and natural language processing. In^9^, GRU is compared directly to LSTM using the Yelp review dataset, showing that GRU performs faster and maintains comparable accuracy, making it well-suited for real-time edge scenarios^10^. identifies various optimization techniques to train Recurrent Neural Networks (RNNs), e.g., pseudo-Newton methods as well as targeted initialization approaches, testing their efficacy on speech recognition and video captioning tasks.

Logistic Regression (LR) remains an underlying classification method, particularly where interpretability is crucial. The study in^11^ discusses its application in imbalanced datasets and highlights improvements such as prior correction and weighting, making it well-suited for binary classification problems in network traffic analysis. In a related domain^12^, surveys ML-based Wi-Fi indoor localization systems using RSSI fingerprinting, exploring preprocessing techniques and architectures like Random Forests, CNNs, and RNNs.

Supervised classification also plays a central role in intrusion detection. A broad review in^13^ compares logic-based, instance-based, Bayesian, and ensemble learning methods, stressing that no single classifier dominates across all scenarios. The value of ensemble learning and careful data preparation is emphasized. Additionally^14^, explores wireless device classification based on environmental context (indoor vs. outdoor) using RF signals. Ensemble models, particularly Random Forest, achieve high classification performance, aided by robust signal preprocessing.

Studies comparing LSTM and GRU continue to show practical trade-offs in performance and efficiency. The study in^15^ investigates feedforward neural networks for RSSI modeling, identifying that training configurations like ReLU activation and Adam optimizer lead to better positioning accuracy. RNNs further benefit from architectural innovation and optimized training methods^16^.

The review in^17^ underscores the importance of tuning supervised ML algorithms for effective classification and advises a hybrid strategy for tackling high-dimensional or heterogeneous datasets. Similarly^18^, examines ML-driven indoor positioning, emphasizing the limitations of GPS indoors and the promise of ML-enhanced localization techniques using RSSI, TOA, and POA.

The integration of ML into intrusion detection is further supported by^19^, where multiple classifiers including SVM and Naive Bayes are evaluated for detecting Deauthentication DoS attacks with high accuracy. In practical deployments, ensemble algorithms like Random Forest and XGBoost consistently perform well in both classification and regression tasks^20,21^. XGBoost, in particular, outperforms traditional Random Forest by leveraging gradient boosting and efficient parallelization^22^. Recent deep-learning NIDS research combines a sequential DNN with Extra-Trees feature selection on UNSW-NB15, reporting ≈ 97.9% binary accuracy while noting future work on multiclass detection and leaving real-time inference characteristics largely unspecified^23^.

Beyond IDS, XGBoost has been used for telecom forecasting and traffic prediction^24^ and shown strong classification results for encrypted and peer-to-peer flows^25^. Indoor localization in non-line-of-sight (NLOS) conditions is tackled in^26^, which compares RF and XGBoost, finding both effective but XGBoost slightly superior. Random Forests continue to be favored for their ease of tuning, robustness to overfitting, and adaptability across domains^27–29^.

Adaptive Random Forest (ARF), an enhancement of the traditional RF, is introduced in^30^, where tree performance is dynamically weighted, improving classification and regression accuracy in streaming and dynamic environments. Meanwhile, Support Vector Machines are explored in^31^ for face recognition, diagnosis, and network modeling due to their strong generalization capabilities rooted in statistical learning theory.

Advanced SVM use is demonstrated in^32^, where decision-making in hybrid LiFi–WiFi networks is optimized using neighboring channel conditions. SVMs also support Wi-Fi traffic prediction in^33^, where they outperform traditional forecasting models using error metrics like MSE and NMSE. Simpler yet effective approaches like kNN are reviewed in^34^, with extensions to classification and regression tasks, especially when input data is poorly structured.

KNN-based localization techniques remain highly relevant. The study in^35^ refines KNN for indoor positioning using optimized fingerprinting strategies, while^36^ introduces a weighted KNN variant that balances RSS similarity and spatial distance to enhance precision. Deep Neighborhood Learning (DNL) in^37^ builds on this by modeling RSS fingerprints as graphs, using graph neural networks to capture contextual relationships between access points and signal patterns.

Traditional classifiers still hold ground in anomaly detection and throughput modeling. In^38^, various ML methods are applied to wireless data for classification and optimization. These include decision trees, kNN, and boosting methods for robust and fast detection of anomalies. Foundational techniques like linear regression and Random Forest are compared in^39^ for their ability to model relationships between signal metrics and throughput.

The study in^41^ explores kNN-based classification in noisy environments, identifying variants that improve robustness and generalization. Similarly^42^, applies ML techniques to uncover relationships in Wi-Fi datasets. Random Forest outperforms linear regression in predicting round-trip time (RTT), revealing it as a key predictor of Wi-Fi throughput. Complementing these findings, a lightweight IDS that uses simple statistics-based feature selection with classical ML (e.g., LR, SVM, RF) achieves competitive accuracy on IoTID20 and ≈ 99%+ on NSL-KDD while reducing computation time, highlighting the suitability of compact models for edge devices^43^.

While the literature presents a broad range of machine learning models and techniques for Wi-Fi security, most existing approaches either emphasize high-performance, cloud-based solutions or overlook the importance of deployment in real-time, low-cost edge environments. Few works address the trade-off between accuracy and interpretability, which is critical in embedded systems and IoT applications. The present study addresses this gap by introducing a lightweight, deployable Wi-Fi monitoring framework that integrates deep temporal learning with explainable models. Leveraging NodeMCU-based packet sniffing, OLED-based real-time visualization, and hybrid architectures combining LSTM, GRU, and RNN with Logistic Regression, this work offers a scalable and interpretable solution tailored for edge-level Wi-Fi anomaly detection.

Table 1 summarizes our proposed work and other works, including methodologies, objectives, dataset, and results of various machine learning models. It shows LSTM and GRU’s ability in handling sequential data, where GRU is faster and LSTM is superior in accuracy. GRU variants are shown to reduce complexity while maintaining comparable results. The table also reviews other algorithms, such as SVM, Random Forest, and XGBoost, noting their strengths in classification tasks and applications ranging from speech recognition to Wi-Fi traffic prediction. The proposed work builds on these studies by using AI to assess Wi-Fi performance and identify network threats in real time.

**Table 1:** Comparison between the proposed work and other works.

Ref#	Methodology	Objective	Dataset used	Models & Results	Limitation
^3^	DNN with Extra Tree feature selection on UNSW-NB15; data standardized, split, and reduced to 8 features using ReLU	Develop a Sequential DNN IDS with ReLU and feature selection on UNSW-NB15 for accurate, efficient, and interpretable attack detection.	UNSW-NB15	Stats-based feature selection + DT, RF, AdaBoost >99.9% accuracy on IoTID20, 27–63% faster training.	IoT constraints, manual feature selection complexity, and no discussion of overfitting or computational limits.
^9^	Compare LSTM and GRU as methods for deep learning models, evaluating performance based on dataset size, text length, and five quantitative indicators.	Compare performance differences between LSTM and GRU	Yelp	GRU is 29.29% faster than LSTM when processing the same dataset	LSTM and GRU address short-term memory issues, but have their complexities
^13^	Logic-based techniques, Perceptron-based techniques, and the Bayesian Networks method for supervised classification	Improve classification accuracy through ensemble methods.	-	C4.5 is recognized for its effective balance between error rate and speed, making it an efficient and reliable decision tree algorithm.	Handling noise and missing feature values, and mitigating the risks associated with unsuitable data collection methods.
^14^	Custom Android app, SigCap, to collect GPS, Wi-Fi, 4G, and 5G data	Develop an automatic indoor/outdoor classification method using RF signals	212,770 collected samples	Random Forest achieved an accuracy of more than 99% and an F1-score.	Inaccurate classification of indoor/outdoor environments
^17^	Test different machine learning algorithms, including SVM, on the Diabetes dataset to find the most accurate model.	Compare various supervised machine learning algorithms for classification efficiency.	uses multiple datasets	SVM was noticed as the most precise and accurate algorithm.	K-NN’s sensitivity to irrelevant features, inefficiencies in NN, decision tree limitations with diagonal partitioning
^22^	Comparing (XGBoost), (RF), and gradient boosting by evaluating both tuned and default settings.	Select a better default parameterization and explore alternatives for XGBoost grids.	28 different datasets sourced from the UCI	Tuned gradient boosting was the best performer in 10 out of 28 datasets, while tuned XGBoost excelled in 8 datasets.	Excessive computational and overfitting during parameter estimation
^23^	DNN + Extra Tree feature selection on UNSW-NB15; standardized, split, reduced to 8 features with ReLU	Deep learning IDS with Sequential DNN + ReLU + feature selection on UNSW-NB15 for accuracy, efficiency, and interpretability.	UNSW-NB15	Sequential DNN + ReLU + Extra Tree feature selection; ≈97.9% binary accuracy.	Focused on offline evaluation, it lacks real-time inference metrics and deployment constraints.
^29^	Uses logistic regression with the glm function, CART analysis with ctree, and random forest with the randomForest function	Evaluate random forest and logistic regression methods for binary classification problems.	22,151 collected samples	Random forest outperforms logistic regression in accuracy and effectively ranks variable importance.	Regularized regression struggles with nonlinear relationships; imbalanced data affects accuracy and increases cost.
^33^	Using (SVM) for one-step-ahead and multi-step-ahead traffic prediction in WLANs.	Develop an SVM-based one-step-ahead prediction for WLAN traffic	-	SVM demonstrated high prediction accuracy for WLAN traffic, both in the short term and long term, outperforming other prediction models.	Long-range dependent network traffic complicates the development of effective predictors
^34^	Using the k-Nearest Neighbor (kNN) algorithm for classification and regression tasks	Improve classification accuracy for marginal data outside typical regions	-	Further research is needed to improve classification accuracy for marginal data outside typical regions	High computational cost and slow prediction phases
^43^	Uses statistics-based feature selection with classical ML (DT, RF, AdaBoost); data binarized and reordered before classification.	Build a lightweight IDS leveraging statistical feature selection and classical ML for efficient IoT intrusion detection.	IoTID20 and NSL-KDD	Statistics-based feature selection + DT/RF/AdaBoost; >99.9% accuracy on IoTID20, 27–63% faster training.	IoT device constraints and complexity of manual feature selection; no discussion of overfitting or computational limits.
Proposed work	Capture network parameters via ESP8266 and classify risks using AI.	Assess Wi-Fi performance using RSSI, deauthentication packets, and noise.	5,600 collected samples	Accurately identified performance issues and security threats.	Restricted OLED display capacity and potential inaccuracies in parameter measurement.

## Methodology

**Fig. 1:**
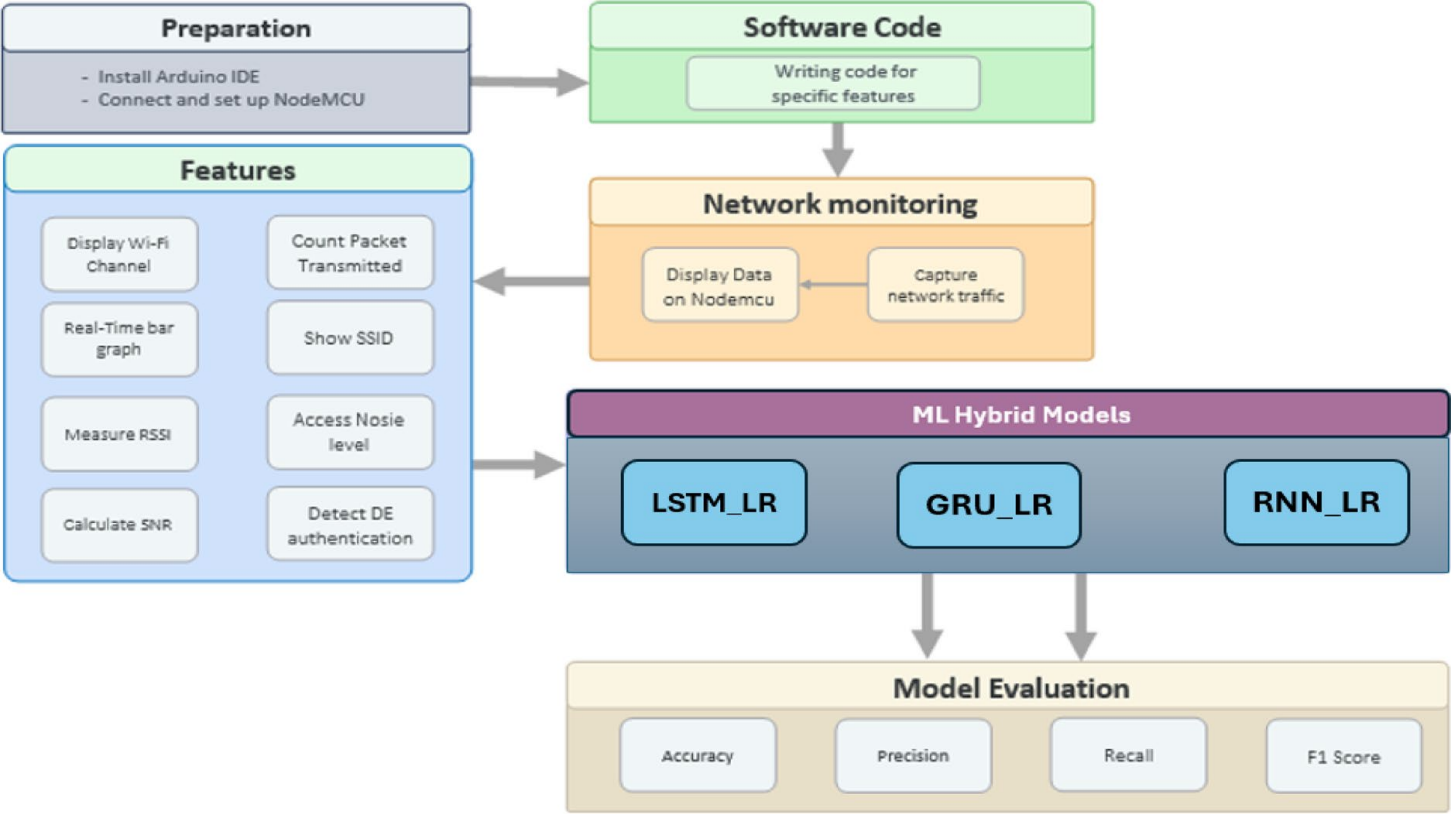
Workflow of the proposed work.

This section shows the comprehensive process involved in the development of a real-time Wi-Fi anomaly detection system utilizing the NodeMCU ESP8266 microcontroller. As depicted in Fig. 1, the process encompasses hardware configuration, software integration, real-time monitoring, hybrid model classification, and performance assessment.

The process commences with the installation of the NodeMCU in conjunction with the Arduino IDE and requisite libraries, such as ESP8266WiFi and Adafruit SSD1306. The NodeMCU has been successfully interfaced via Micro-USB, and custom firmware has been programmed to enable Wi-Fi packet sniffing and the generation of deauthentication packets, in conjunction with the Adafruit GFX library. When turned on, the microcontroller performs sweeps of nearby networks and records data such as SSID, channel, RSSI, SNR, noise, and the count of deauthentication packets. All data that is gathered is displayed in real-time through an OLED display with an I2C interface.

Key functional modules include monitoring packet data transmission, assessing signal quality and noise, channel probing, and displaying suspicious traffic. The minimalist setup facilitates real-time monitoring in resource-constrained environments without necessitating extensive infrastructure.

For anomaly detection purposes, the collected data is transmitted to a set of hybrid machine learning models: LSTM_LR, GRU_LR, and RNN_LR. These models blend the temporal learning capability of deep neural networks (LSTM, GRU, RNN) with the efficiency and interpretability of Logistic Regression (LR). Specifically, LSTM is employed to capture long-term dependencies, GRU provides a lighter variant with comparable accuracy, and RNN serves as the temporal baseline. Logistic Regression is the decision layer for efficiency and interpretability improvements.

This hybrid architecture augments anomaly detection by combining temporal observations with explicit classification rules, rendering it well-suited for detecting attack patterns such as bursts of de-authentication packets or declines in signal quality.

To mitigate the inherent challenge of class imbalance within the dataset, a two-pronged strategy was employed. First, class weighting was implemented for all traditional machine learning models; specifically, the class_weight=’balanced’ parameter was used to adjust the influence of each sample during training inversely proportional to its class frequency. Second, stratified sampling was applied throughout the experimental pipeline to approve that the original class distribution was preserved in all training and testing subsets.

### Model workflow

**Fig. 2:**
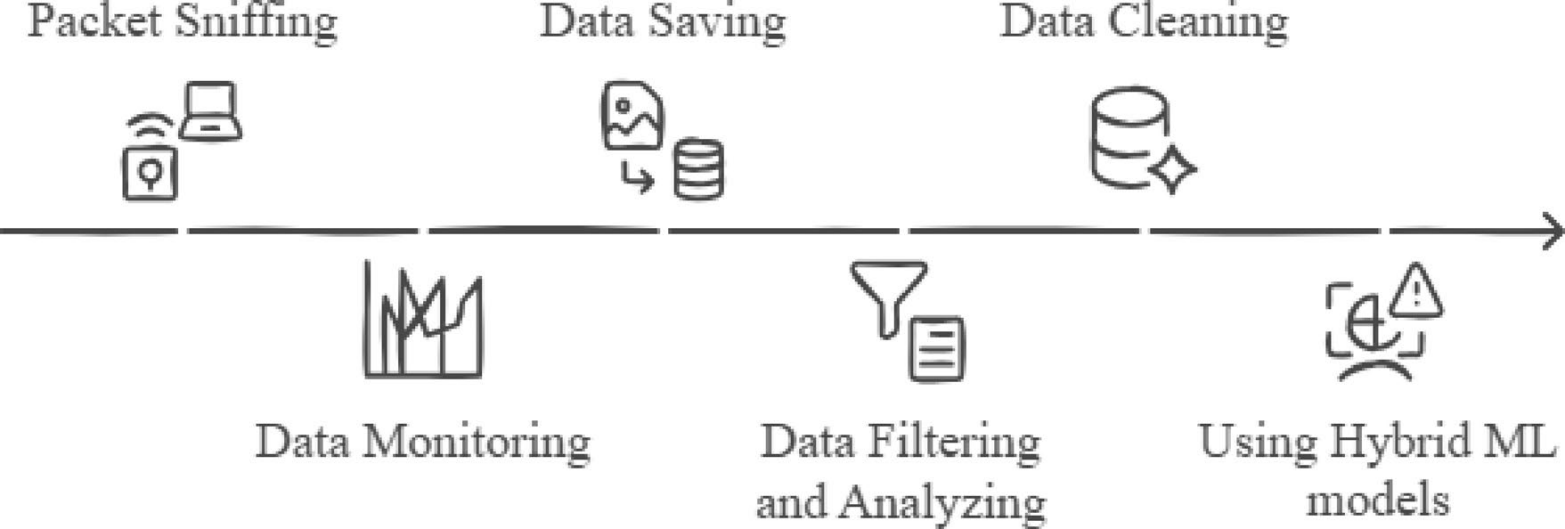
Overview of model architecture.

The overall workflow of the proposed Wi-Fi monitoring framework is illustrated in Fig. 2. The procedure has six successive steps that all contribute to the conversion of raw network traffic to actionable intelligence using hybrid machine learning models:

1. Packet Sniffing: The process starts by installing the NodeMCU (ESP8266) microcontroller, flashing it with Wi-Fi packet sniffing-supported firmware. The device is connected to a laptop to begin capturing live traffic from nearby wireless networks.
2. Data Monitoring: Received Packets are graphed live on an OLED screen via I2C. Key signals such as RSSI, SNR, Wi-Fi channel, and deauthentication packet count are plotted, allowing out-of-pattern activity or interference to be identified simultaneously.
3. Data Saving: To support long-term analysis, the data recorded is stored in structured formats such as Excel or CSV. CoolTerm-like applications are used to interact with the NodeMCU and download the traffic logs for offline analysis and model training.
4. Data Filtering and Analysis: In this stage, the key features are selected from the dataset to evaluate network performance and detect security threats. Performance metrics like deauthentication packet frequency and SNR trends are used to identify potential intrusions. In this study, a total of 5,653 samples from the dataset were prepared for model evaluation.
5. Data Cleaning: Data cleaning is performed before training to remove inconsistencies, outliers, and unwanted noise from the data. This improves model accuracy and provides good feature representation for machine learning analysis.
6. Applying Hybrid AI Models: Finally, hybrid models combining deep learning and traditional classification are applied to the cleaned dataset. Especially, three architectures LSTM_LR, GRU_LR, and RNN_LR are used. The deep learning modules (LSTM, GRU, RNN) can easily capture time-dependent patterns in sequential network data, and Logistic Regression is used as a light decision layer to enhance interpretability and classification accuracy. These models collectively enable accurate anomaly detection and behavioral classification in real-time Wi-Fi environments.

### Packet sniffing

Packet sniffing is accomplished with the NodeMCU (ESP8266) microcontroller used in promiscuous mode that enables the device to capture all Wi-Fi packets that are broadcast on a selected channel irrespective of the source or destination. This gives the NodeMCU the capability to operate as a passive listener in the 2.4 GHz frequency range that supports up to 13 channels depending on where it is located.

The process begins with selecting a target channel via sequential scanning or plugging the device into a specific one for targeted monitoring. When activated, the NodeMCU takes snapshots of a number of 802.11 frame types such as management frames (e.g., beacon, probe), control frames (e.g., ACK, RTS/CTS), and data frames carrying payload traffic. This raw traffic data contains useful information about the topology and activity of nearby networks and is the basis for subsequent feature extraction and anomaly detection.

### Data monitoring

In addition to enabling real-time network awareness, the NodeMCU platform features an OLED screen that displays a set of parameters indicative of performance and security status. Parameters that include real-time bar graph depiction of network traffic, the current operating channel of Wi-Fi, the number of packets sent, and the DA packet detection rate are all included. It also provides important wireless parameters such as the Service Set Identifier (SSID), Received Signal Strength Indicator (RSSI), noise level in the environment, and Signal-to-Noise Ratio (SNR). All these parameters together allow users to monitor link quality continuously, detect abnormal fluctuations, and detect likely security attacks with low latency.

### Data preparation

**Fig. 3:**
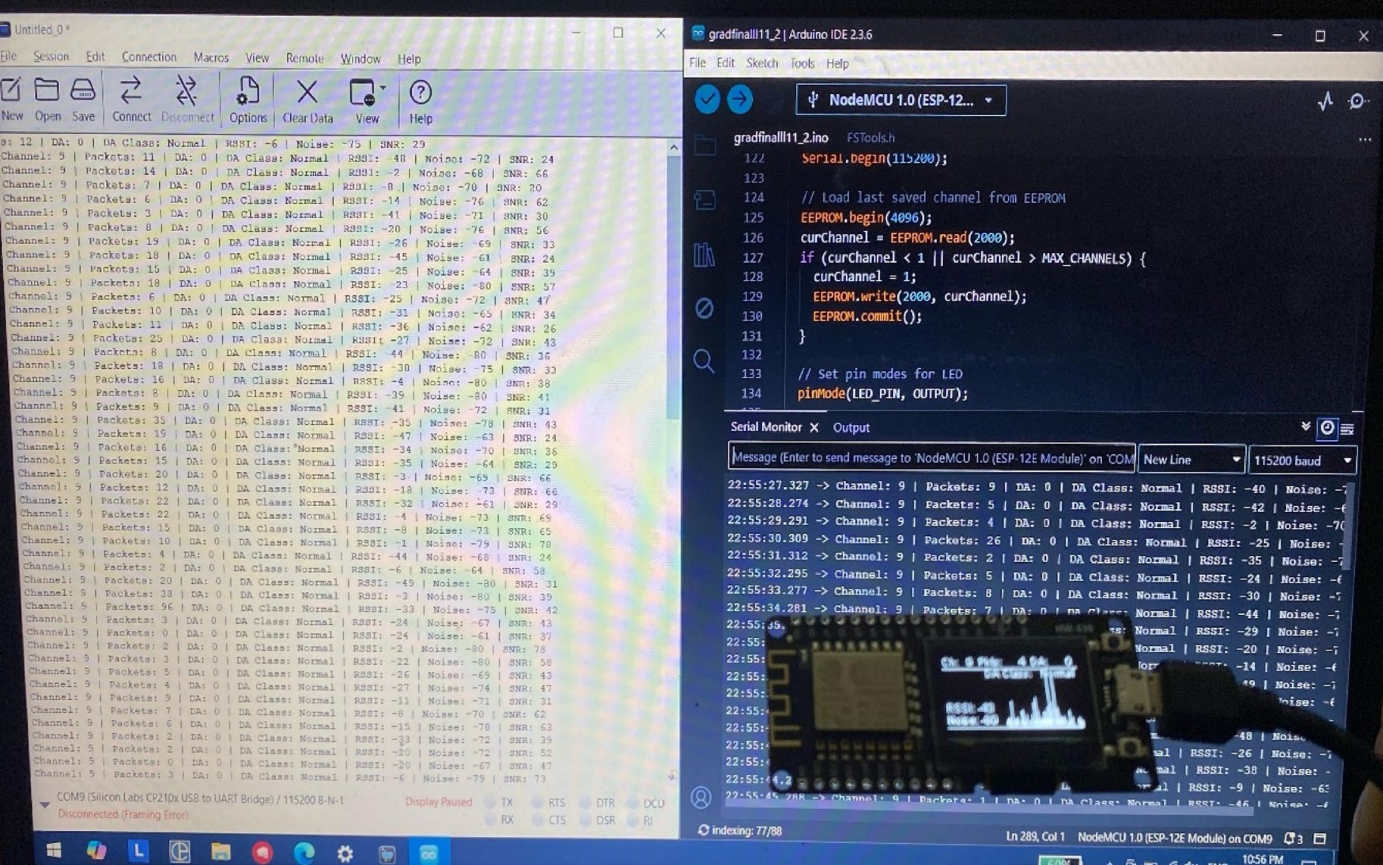
Data logging setup using NodeMCU, Arduino IDE, and CoolTerm.

Figure 3 shows the practical setup used during the data collection phase of the proposed work. ESP8266 NodeMCU serial output is displayed in real time by using the Arduino IDE Serial Monitor. For offline analysis and persisting data, the CoolTerm terminal software was used to capture the serial output and save it in a CSV file. This method enabled the seamless logging of key network metrics, ensuring consistency across the more than 5,600 collected samples. The image also showcases both the coding environment used for firmware development and the OLED display output, effectively illustrating the integration between software implementation and hardware-level data visualization. Data Specifications.

**Fig. 4:**
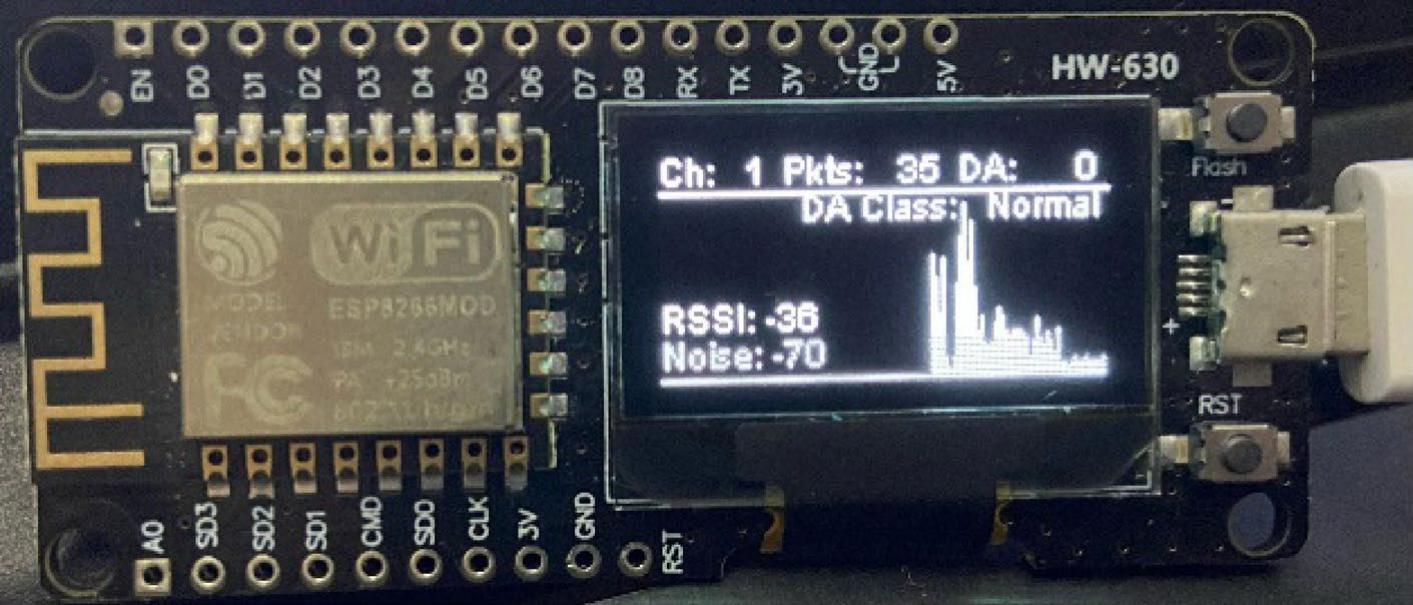
Data displayed on OLED.

**Fig. 5:**
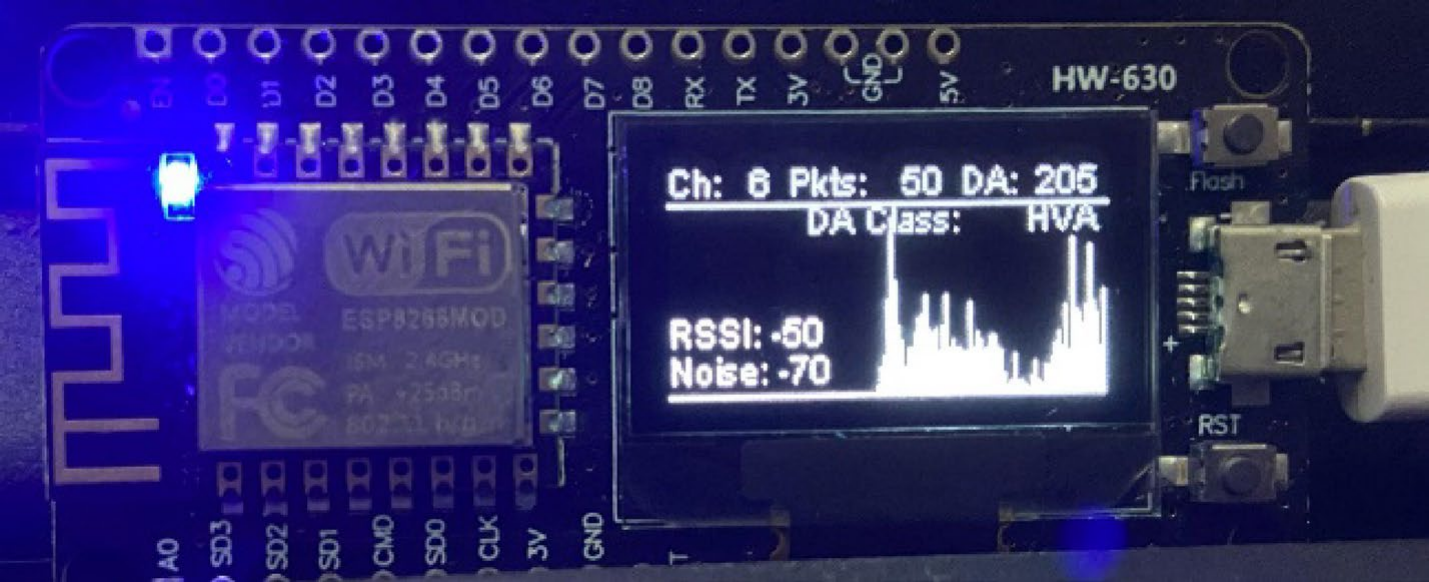
Real-time visualization on OLED.

Figures 4 and 5 show real-time visualizations of network activity via an OLED display mounted on the NodeMCU. The interface features a dynamic bar graph that plots packet density over time, providing key statistics such as the Wi-Fi channel, number of packets sensed, Received Signal Strength Indicator (RSSI), noise reading, and Deauthentication (DA) packet count.

Received Signal Strength Indication, or RSSI, is the power received in decibels relative to a milliwatt (dBm) and needs to be employed to estimate proximity of the device and signal quality. Signal-to-Noise Ratio (SNR) can be computed as:

1 $$\:\text{S}\text{N}\text{R}\:\left(\text{d}\text{B}\right)=\text{R}\text{S}\text{S}\text{I}\:\left(\text{d}\text{B}\text{m}\right)-\text{N}\text{o}\text{i}\text{s}\text{e}\:\text{L}\text{e}\text{v}\text{e}\text{l}\:\left(\text{d}\text{B}\text{m}\right)$$

An increased SNR means stronger and more stable communication, which has a direct relation to data throughput and connection stability.

It also monitors the Wi-Fi channel (1–13 within the 2.4 GHz band) and aggregate packet traffic, providing a general idea of the activity underway. Of special concern is the count of deauthentication packets (DA), which are early warning indicators of potential denial-of-service (DoS) attacks. For instance, Fig. 5 shows DA = 205, indicating a potential high-level threat.

To contextualize these values, Table 2 outlines a classification scheme for DA activity, mapping the rate of deauthentication packets to corresponding security threat levels. This classification aids both in feature labeling for supervised learning and in the real-time identification of network anomalies. In addition to DA thresholds, RSSI ranges were also integrated to provide a more robust classification of threat levels. The classification thresholds for deauthentication (DA) packet rates and RSSI values in Table 2 were determined through a combination of empirical analysis and established wireless security principles. A dataset of over 5600 network parameter entries was collected from controlled experiments simulating both normal Wi-Fi operation and varying intensities of deauthentication attacks. Statistical analysis of DA packet rate distributions under benign and attack conditions revealed distinct separation ranges. For example, normal network activity rarely exceeded 40 packets/sec, low-intensity attacks typically ranged from 40 to 80 packets/sec, and sustained high-volume attacks produced rates well above 150 packets/sec. In addition to DA frequency, we also considered the duration of device disconnection as a key factor in classification: normal scenarios were associated with 0–1 s of disconnection, low-volume attacks typically resulted in 1–20 s, moderate attacks in 20–60 s, and high-volume attacks in disconnections exceeding 60 s. This dual-criterion approach (packets/sec and disconnection time) ensured that thresholds aligned not only with packet-level anomalies but also with the real impact on connectivity. This procedure is compatible with prior work demonstrating that DA frequency is an effective indicator for classifying attack severity and that thresholds should be empirically tuned to the deployment environment^44^. Similarly, RSSI thresholds were informed by widely accepted Wi-Fi performance categorizations, where RSSI ≥ − 50 dBm indicates strong connectivity, RSSI < − 60 dBm represents moderate to weak connectivity, and RSSI < − 70 dBm is associated with poor or unstable links^45^. The final ranges presented in Table 2 reflect both the observed behavior in our dataset and the recommendations from relevant literature. A custom code function was developed to dynamically link RSSI values to their corresponding DA packet rates, allowing for real-time multi-parameter threat classification. This dual-condition scheme enhances the system’s ability to detect and respond to network anomalies in dynamic wireless environments while remaining lightweight and interpretable for embedded deployment.

**Table 2:** Classification of Wi-Fi activity based on deauthentication rate and RSSI signal Strength.

Classes Representation for ML	Classification	Description	Classification condition for DA	Classification condition for RSSI
0	Normal	No unusual or suspicious activity was observed	DA ≤ 40 packets/sec	RSSI ≥ −50 dBm
1	LVA (Low-Level Malicious Activity)	Minor security concerns or potential vulnerabilities.	40 < DA ≤ 80 packets/sec	−60 ≤ RSSI < −50 dBm
2	MVA (Medium-Level Malicious Activity)	Moderate level of security risk.	80 < DA ≤ 150 packets/sec	−70 ≤ RSSI < −60 dBm
3	HVA (High-Level Malicious Activity)	Significant and active security threat.	DA > 150 packets/sec	RSSI < −70 dBm

### Machine learning mechanisms

Hybrid machine learning models combine deep learning with traditional classifiers to provide more precise, efficient, and interpretable predictions. Three hybrids were used in the study: LSTM_LR, GRU_LR, and RNN_LR. Deep learning components (LSTM, GRU, and RNN) can learn efficient temporal patterns in Wi-Fi traffic, while Logistic Regression (LR) simplifies the last-stage classification and enables model interpretability.

This design leverages the benefits of sequential data modeling and lightweight decision-making, making it appropriately suited for real-time anomaly detection on resource-constrained devices. After the dataset was extracted via NodeMCU and cool term, the cleaning, preprocessing and the running of the ML models were all done on a Dell Precision system equipped with an Intel Core i7 processor (2.9 GHz), 16 GB of RAM, and an NVIDIA Quadro M1200 graphics unit with 4 GB of dedicated memory. The following subsection explains every model.

#### Hybrid LSTM with LR

The LSTM_LR model combines Long Short-Term Memory (LSTM) networks and Logistic Regression (LR) to leverage the temporal learning capabilities of deep models with the interpretability of linear classifiers^40^. LSTM is particularly well-suited to learn sequences, and thus would be employed to capture time-based Wi-Fi traffic patterns. Instead of a dense neural output layer, the final state of the LSTM is passed directly to a logistic regression component. This design improves computational efficiency and readability, enabling real-time deployment on low-resource devices.

**Fig. 6:**
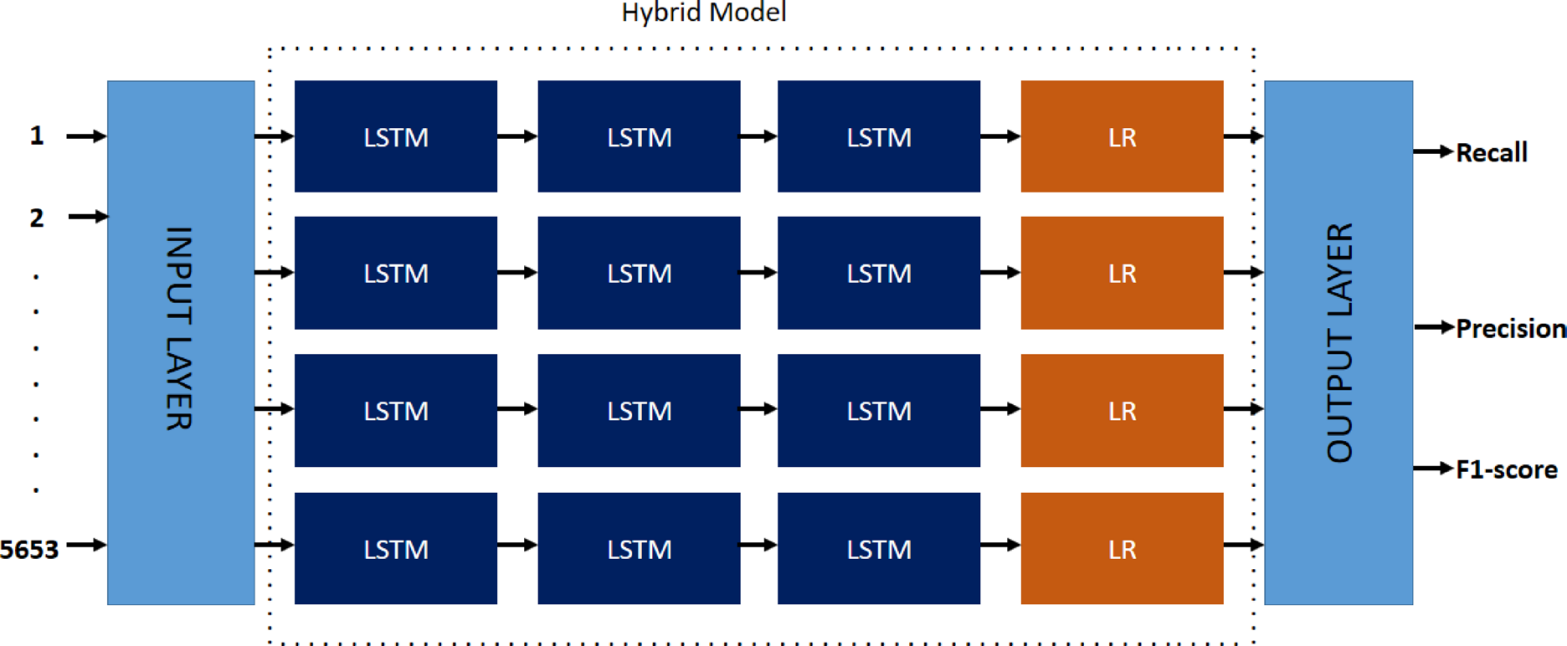
Hybrid model between LSTM and LR.

As shown in Fig. 6, the design consists of LR classifiers and stacked LSTM layers. The model produces standard evaluation metrics like F1-score, precision, and recall. Training is performed using a binary cross-entropy loss function.

2 $$\:\mathcal{L}\left(\theta\:\right)=-\left[\sum\:_{i=1}^{N}{y}^{\left(i\right)}\text{log}\left(\sigma\:\left({w}^{T}{h}_{T}^{\left(i\right)}+b\right)\right)+(1-{y}^{\left(i\right)})\text{log}\left(\sigma\:\left({w}^{T}{h}_{T}^{\left(i\right)}+b\right)\right)\right]$$

- $\:N$: Total number of training samples.
- $\:{y}^{\left(i\right)}$: Ground truth label for the $\:{i}^{th}$ sample.
- $\:\sigma\:$: Sigmoid activation function.
- $\:w$: Weight vector of the logistic regression layer.
- $\:{h}_{T}^{\left(i\right)}$: Final hidden state from the LSTM for the $\:{i}^{th}$ input.
- $\:b$: Bias term in the logistic regression layer.

#### Hybrid GRU with LR

The GRU_LR hybrid model combines Gated Recurrent Units (GRUs) with Logistic Regression (LR) to strike a balance between temporal pattern recognition and interpretability. GRUs, which are recurrent neural networks, are best suited for pulling sequential patterns from time-series data and offering a computationally less expensive alternative to LSTMs. The final hidden state output of the GRU is passed to a logistic regression layer after training for classification^46^.

This approach enhances performance in tasks such as anomaly detection and time-based classification while keeping the decision process explainable and straightforward. The model is optimized using a binary cross-entropy loss function:

3 $$\:L=-\sum\:_{i=1}^{N}\left({y}_{i}\text{l}\text{o}\text{g}\left(\sigma\:\right({W}_{lr}{h}_{t}+{b}_{lr}\right))+\left(1-{y}_{i}\right)\text{l}\text{o}\text{g}(1-\sigma\:\left({W}_{lr}{h}_{t}+{b}_{lr}\right)\left)\right)$$

- $\:{y}_{i}$: Ground truth label for the $\:{i}^{th}$ input.
- $\:{W}_{lr}$: Weight matrix of the logistic regression classifier.
- $\:{h}_{t}$: Hidden state output from the GRU at time step $\:t$.
- $\:{b}_{lr}$: Bias term in the logistic regression classifier.

By combining the GRU’s sequential modeling strengths with LR’s simplicity, this hybrid architecture delivers a strong, interpretable solution suitable for real-time network threat detection.

#### Hybrid RNN with LR

RNN_LR hybrid model integrates Recurrent Neural Networks (RNNs) and Logistic Regression (LR) to leverage the strengths of sequential learning and explainable classification. RNNs are particularly well-suited for capturing temporal relationships in sequential data, such as network traffic over a specific period. The final hidden state of the RNN, used as input to a logistic regression layer, is employed to produce a classification choice based on learned patterns.

This type of architecture proves helpful when both model explainability and temporal context are crucial, such as in network intrusion detection, where understandable alarms are needed.

Forward pass in RNN:

4 $$\:{h}_{t}=f({W}_{h}\cdot\:{h}_{t-1}+{W}_{x}\cdot\:{x}_{t}+{b}_{h})$$

- $\:{h}_{t}$: Hidden state at time $\:t$.
- $\:{h}_{t-1}$: Hidden state from the previous time step.
- $\:{x}_{t}$: Input vector at time $\:t.$.
- $\:{W}_{h},{W}_{x}$: Weight matrices for the hidden state and input.
- $$\:{b}_{h}$$: Bias vector.
- $\:f$: Activation function (Tanh or ReLU).

Logistic Regression Layer (Output Layer):

5 $$\:y=\sigma\:({W}_{o}\cdot\:{h}_{T}+{b}_{o})$$

- $\:{h}_{T}$: Final hidden state of the RNN.
- $\:\sigma\:$: Sigmoid activation function.
- $\:{W}_{o}$: Weight matrix for logistic regression.
- $\:{b}_{o}$: Bias term in the logistic regression layer.

Loss Function:

6 $$\:L=-\frac{1}{N}\sum\:_{i=1}^{N}({y}_{i}\cdot\:\text{log}\left({\widehat{y}}_{i}\right)+(1-{y}_{i})\cdot\:\text{log}(1-{\widehat{y}}_{i}))$$

- $\:{\widehat{y}}_{i}$: Predicted probability for the $\:{i}^{th}$ sample output from the logistic regression layer.

By pairing RNN with LR, this hybrid model offers a lightweight and interpretable structure while still effectively capturing sequential trends for accurate classification.

### Evaluation metrics

To assess the performance of each hybrid model, we utilized standard evaluation metrics, as detailed below.

#### Accuracy

Accuracy in machine learning measures the proportion of correctly classified instances among the total instances. It is a key performance metric, especially for balanced datasets, but may be misleading in imbalanced scenarios, where precision, recall, and F1-score provide a more comprehensive evaluation of performance.

7 $$\:Accuracy=\:\frac{correct\:prediction}{total\:prediction\:}\times\:100$$

#### Recall

The key evaluation metric for classification models is recall, especially when false negatives are more critical than false positives. It is defined as the proportion of actual positive cases that the model correctly identifies.

8 $$\:Recall=\frac{True\:positive}{True\:positive+False\:negative}$$

#### Precision

Precision in machine learning quantifies the proportion of true positive predictions among all positive predictions made by the model. It is crucial in scenarios where false positives carry significant consequences, ensuring that positive classifications are reliable and minimizing incorrect identifications.

9 $$\:Precision=\frac{True\:positive}{True\:positive+False\:positive}$$

#### F1 score

The F1 score is defined as the harmonic means of precision and recall providing a single number that balances both. F1 Score is more useful in the case of imbalanced datasets than AUC-ROC, where one class is heavily dominant than the other. It is useful when both false positives (FP) and false negative (FN) are important as it gives equal weight to precision and recall.

10 $$\:F1=2\times\:\frac{Precision\:\times\:Recall}{Precision+Recal}$$

#### ROC

The Receiver Operating Characteristic (ROC) curve is a way to visualize the accuracy of a binary classifier. It is a plot of the True Positive Rate (TPR) against the False Positive Rate (FPR) at different thresholds.

True Positive Rate (TPR):

11 $$\:TPR=\frac{True\:positive}{True\:positive+False\:negative}$$

False Positive Rate (FPR):

12 $$\:FPR=\frac{False\:positive}{False\:positive+True\:negative}$$

Area Under the Curve:

13 $$\:AUC={\int\:}_{0}^{1}TPR\left(FPR\right)d\left(FPR\right)$$

Table 3 provides a systematic breakdown of all configurable parameters found in the provided hybrid machine learning models for a classification task.

**Table 3:** Comprehensive summary of hyperparameters and configuration settings used in machine learning models used.

Hyperparameters	Value	Description
Learning Rate	0.001	Used in PyTorchSklearn Wrapper
Epochs	20	Used for neural network models in PyTorchSklearnWrapper
Batch size	16	Used in PyTorchSklearn Wrapper
Hidden Dimensions	128	Used for LSTM, GRU, and RNN models
Number of Layers	1	-
Dropout	0.2	Only applied when num_layers > 1
Logistic Regression	max_iter = 500	Increased from default (usually 100–1000) to ensure convergence.
Test Split	test_size = 0.2	20% of the data used for testing.
Cross-Validation	StratifiedKFold(n_splits = 5)	5-fold stratified cross-validation for balanced splits.
GPU Utilization	4G NVADIA Quadro M1200	GPU Utilization during training and inference
Random Forest	n_estimators = 100	Default number of trees.

## Results and discussion

This sub-section illustrates the performance evaluation of the suggested hybrid models by key metrics: accuracy, precision, recall, and F1-score. The metrics reveal each model’s ability to generalize from unseen data, handle class imbalances, and minimize false predictions. The results also reflect the impact of model selection, hyperparameter tuning, and preprocessing. Detailed numerical results and comparative remarks are presented in the following subsections to compare the advantages and disadvantages of each approach and determine the top-performing model for real-time Wi-Fi attack detection.

### Simulation results

**Fig. 7:**
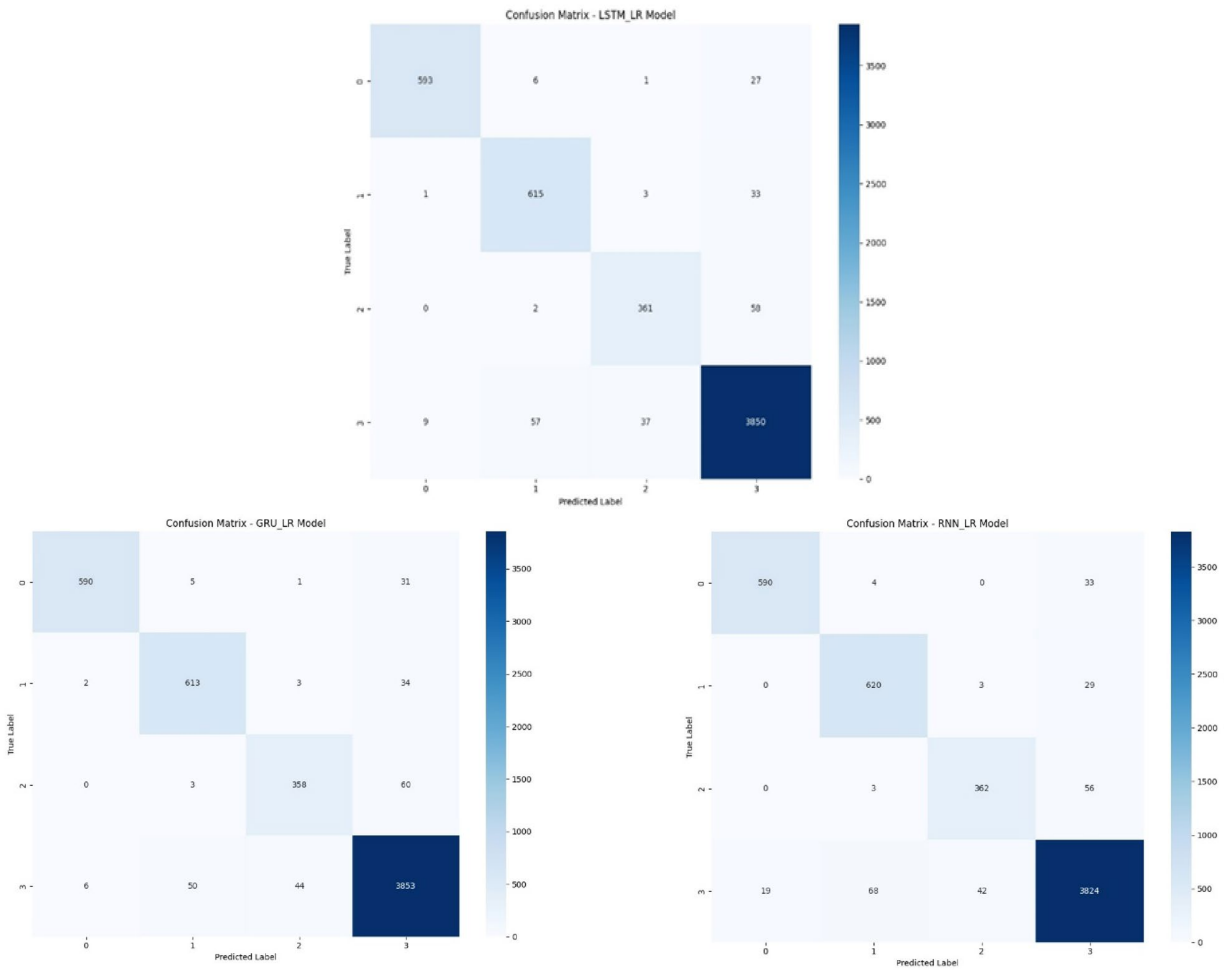
Confusion Matrix of the proposed hybrid models.

Figure 7 shows the confusion matrices of the LSTM_LR, GRU_LR, and RNN_LR models on the four-class classification problem. All three models show great performance on identifying the most dominant class (Class 3), with LSTM_LR correctly classifying 3,850 instances, GRU_LR with 3,853, and RNN_LR being slightly lower at 3,824. The LSTM_LR also performs better on Class 1 (609) and Class 2 (363) owing to its ability to identify long-term dependencies through memory gating mechanisms.

GRU_LR, as lighter in architecture, matches or beats LSTM_LR in Class 1 (613) and Class 2 (368), making it a great option in low complexity and efficiency environments. RNN_LR, despite having promising results in Class 1 (620), lags behind somewhat with Class 0 (506) and Class 2 (348), likely due to the fact that it contains no gating units, which limits its ability to effectively model long-range patterns.

Overall, LSTM_LR and GRU_LR are better than RNN_LR in precision and stability, with stronger classification ability between imbalanced or confusing classes.

**Fig. 8:**
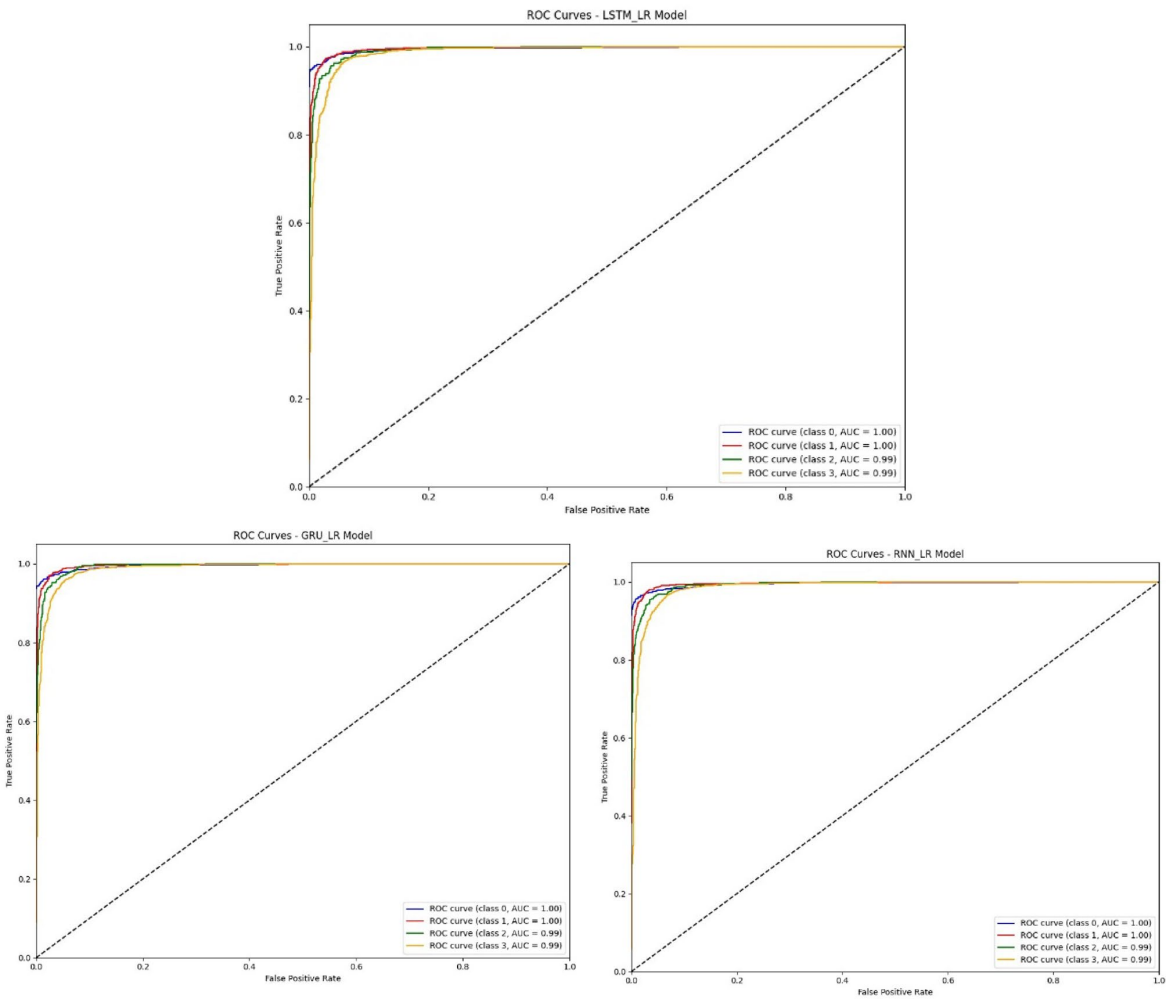
ROC Curves of the proposed hybrid models.

Figure 8 shows Receiver Operating Characteristic (ROC) curves for LSTM_LR, GRU_LR, and RNN_LR models for a four-class problem. All three models are well discriminative with 1.00 Area Under the Curve (AUC) for classes 0 and 1, and 0.99 for classes 2 and 3.This near-ideal performance indicates high sensitivity and specificity with minimal trade-off among true positive and false positive rates.

The LSTM_LR and GRU_LR models have very similar ROC curves, confirming their effectiveness in learning temporal patterns for multi-class classification. The RNN_LR model, while less sharp in curve gradient, still achieves comparable AUC values, proving its dependability even without gated memory units. These consistently high AUC values from all models confirm the performance of the hybrid deep-learning classifiers and prove them suitable for real-time, multi-class Wi-Fi threat detection.

**Fig. 9:**
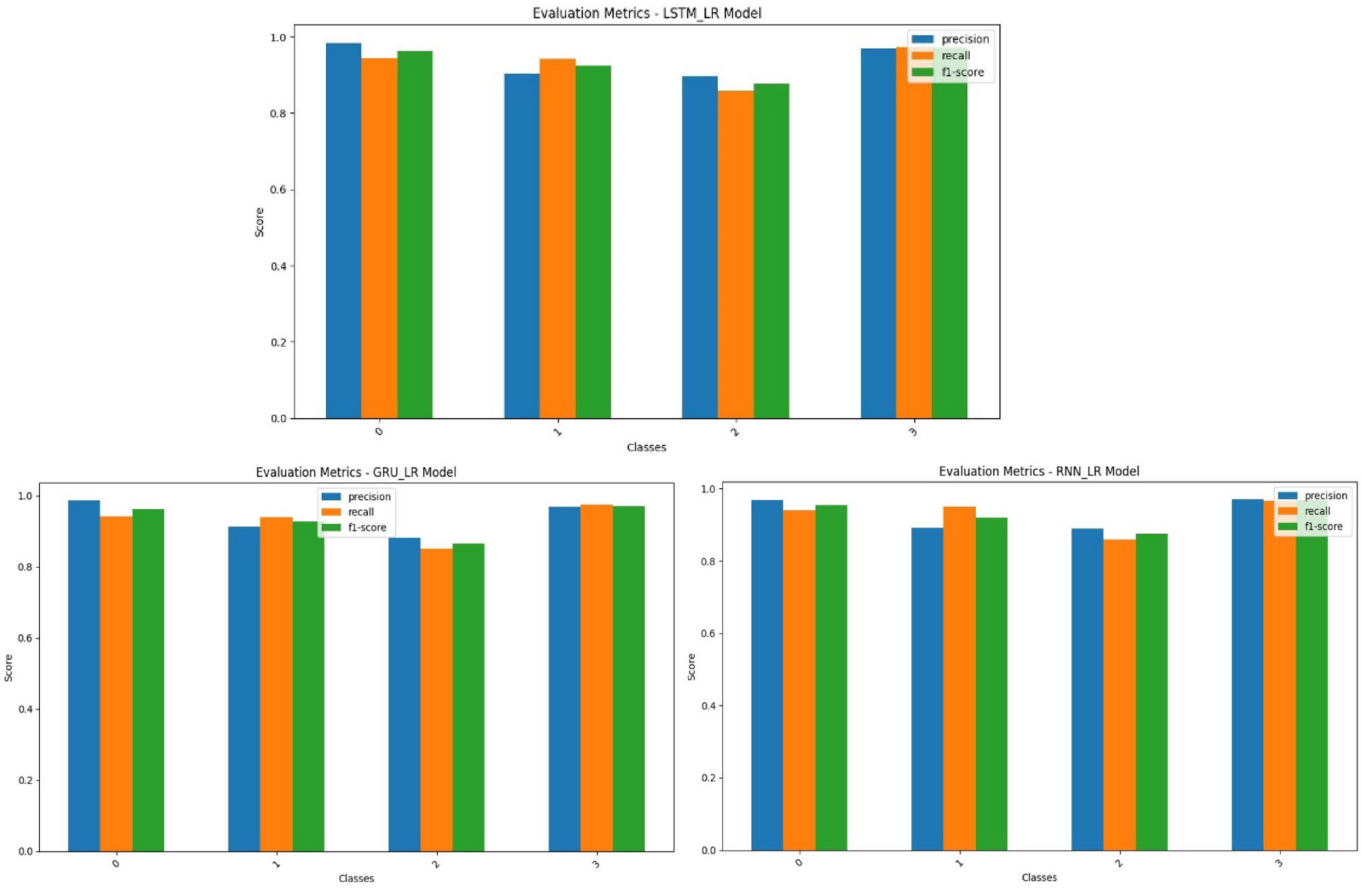
Evaluation Metrics of the proposed hybrid models.

Figure 9 shows the precision, recall, and F1-scores for each class in the three hybrid models: LSTM_LR, GRU_LR, and RNN_LR. The performance of all the models is good, but there are minor variations in class-wise performance. LSTM_LR performs best in classes 0 and 3, but with slightly reduced recall in class 2. GRU_LR performs most consistently across all classes and measures, with class 0 having almost perfect precision. This implies its parsimony combined with generalization ability. Conversely, RNN_LR, although slightly behind class 2 recall, maintains consistent scores for all classes, reflecting a steady, if less specialized, classification ability. Overall, LSTM_LR and GRU_LR perform more strongly, with GRU_LR offering a good accuracy vs. efficiency balance.

**Fig. 10:**
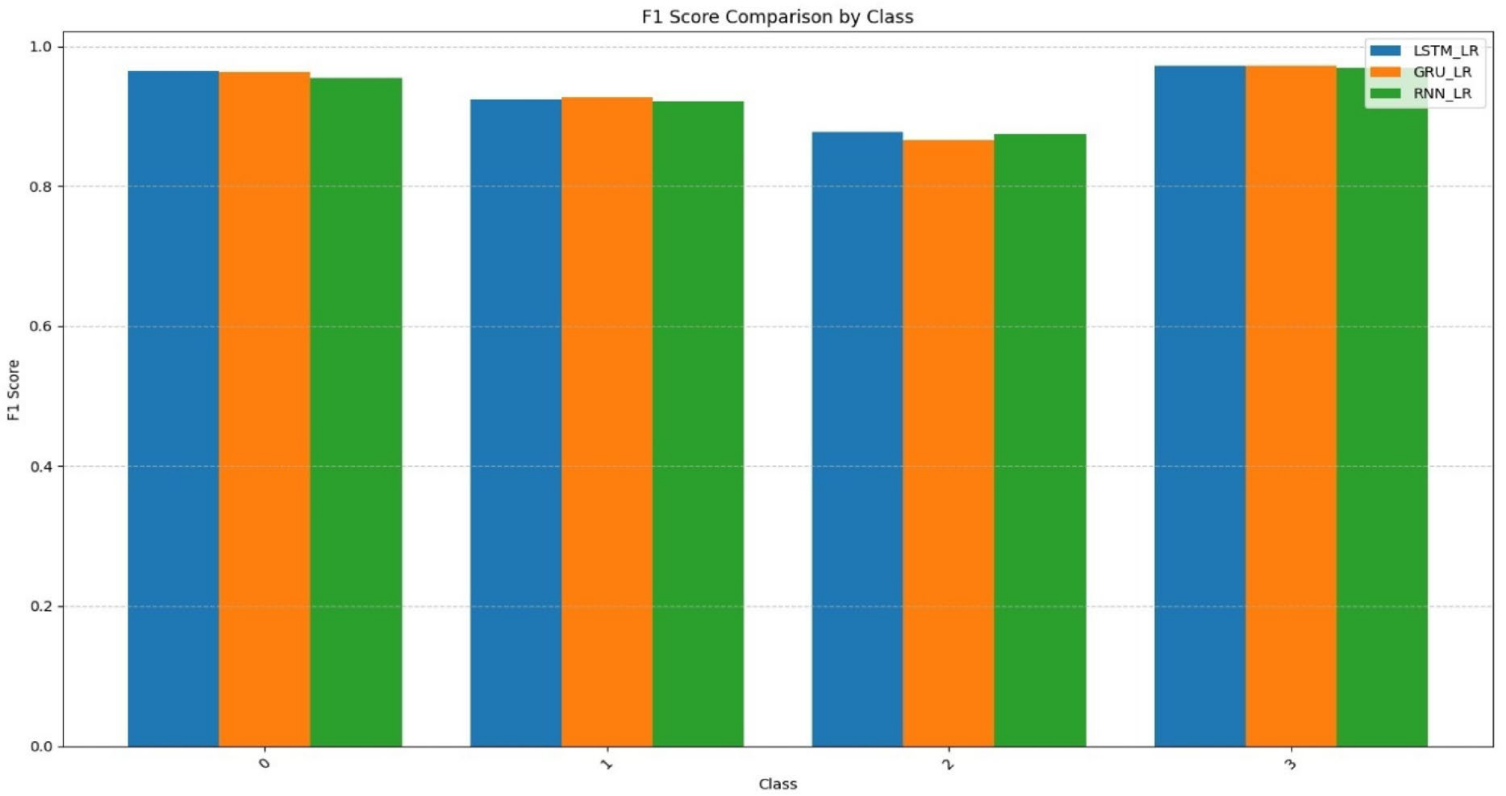
F1-Score Comparison between LSTM_LR, GRU_LR, & RNN_LR.

Figure 10 illustrates the F1-score performance of classes 0 to 3 for the three hybrid models, LSTM_LR, GRU_LR, and RNN_LR. All three models exhibit excellent performance, with all F1-scores above 0.85 for all classes. The highest scores are achieved by classes 0 and 3, where LSTM_LR and GRU_LR marginally outperform RNN_LR, indicating better classification performance for these classes. Whereas Class 2 sees a modest decline in performance for all models, GRU_LR performs the worst in terms of F1-score for the class. LSTM_LR and GRU_LR are both highly comparable trends by class, demonstrating the reliability and strength of the two. Both LSTM_LR and GRU_LR are generally more precise and consistent, and it is important to explain why they work as the model for the multiclass classification task versus the basic RNN_LR model.

**Fig. 11:**
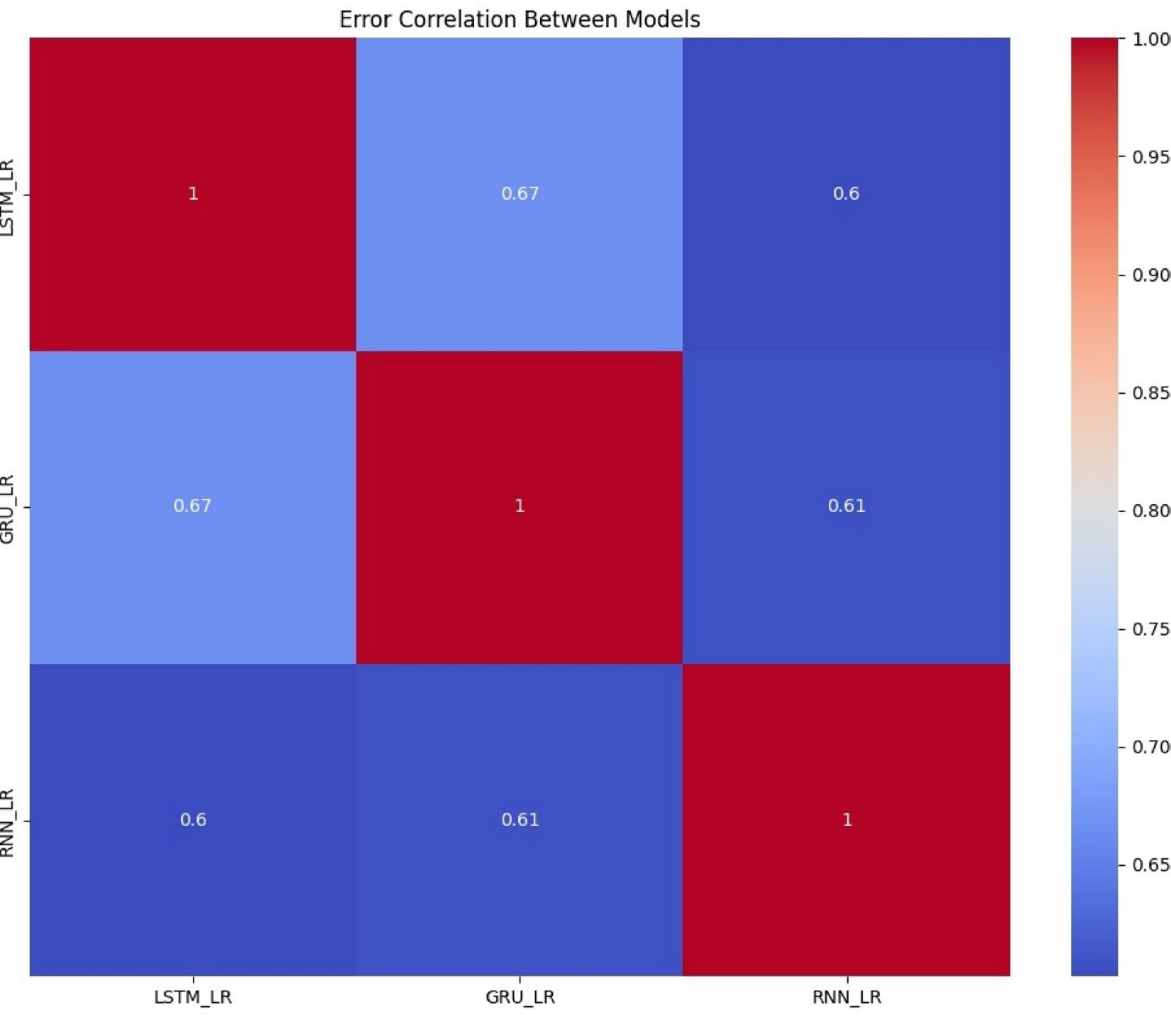
Error Correlation between LSTM_LR, GRU_LR, & RNN_LR.

Figure 11 is a heatmap representing the error correlation between hybrid models LSTM_LR, GRU_LR, and RNN_LR. It is expected that each model will have a perfect self-correlation along the diagonal (value = 1). Among the model pairs, LSTM_LR and GRU_LR have the most significant error correlation (0.67), indicating that they learn very similar temporal behaviors. RNN_LR has lower correlation with LSTM_LR (0.60) and GRU_LR (0.61), indicating more distinct error distributions. They indicate possible complementarity between models, a consideration that is pertinent to ensemble learning or more sophisticated hybrid methods for improved robustness and generalization.

Table 4 summarizes a comparison of precision, recall, F1-score, and overall accuracy for the hybrid models LSTM_LR, GRU_LR, and RNN_LR on a four-class classification task. Both LSTM_LR and GRU_LR achieved the highest accuracy (96%) with stable and uniform performance for all but one class. LSTM_LR maintained a balanced precision and recall, with its peak achieved for Class 3 (F1-score: 0.97). GRU_LR was also, albeit slightly more accurate for Class 0 (0.99), and similarly resilient overall, as LSTM_LR. RNN_LR, while equally effective at 95% accuracy, was slightly less accurate for Class 2 and Class 1. The results highlight the improved generalizability and reliability of LSTM_LR and GRU_LR, particularly for more complex or ambiguous class boundaries. We also ran an additional analysis of the RF model using AUC scores, which ranged between 0.84 and 0.95. These values show that the model can rank instances reasonably well, but it struggles to turn that ranking into accurate final predictions. This weakness is clear in its low precision, recall, and F1-scores as shown in Table 4. In contrast, our hybrid model not only separates classes effectively but also delivers consistent and reliable classification results.

**Table 4:** Comparison of hybrid models results.

	Model & Class	Precision	Recall	F1-Score	Overall Accuracy
**1**	**Hybrid LSTM with LR**				0.96
	Class 0	0.98	0.95	0.96	
	Class 1	0.9	0.94	0.92	
	Class 2	0.9	0.86	0.88	
	Class 3	0.97	0.97	0.97	
**2**	**Hybrid GRU with LR**				0.96
	Class 0	0.99	0.94	0.96	
	Class 1	0.91	0.94	0.93	
	Class 2	0.88	0.85	0.87	
	Class 3	0.97	0.97	0.97	
**3**	**Hybrid RNN with LR**				0.95
	Class 0	0.97	0.94	0.95	
	Class 1	0.89	0.95	0.92	
	Class 2	0.89	0.86	0.87	
	Class 3	0.97	0.97	0.97	
**4**	**Random Forest**				0.76
	Class 0	0.83	0.42	0.56	
	Class 1	0.76	0.16	0.26	
	Class 2	0.89	0.12	0.21	
	Class 3	0.75	0.98	0.85	

## Conclusion

This work explored a real-time Wi-Fi intrusion detection system using hybrid deep learning models deployed on low-cost edge devices. By combining sequential models, such as LSTM, GRU, and RNN, with logistic regression, we aimed to create a lightweight, interpretable, and accurate classification pipeline that utilizes live network features, including RSSI, SNR, packet counts, and deauthentication activity. Across all experiments, the GRU_LR model emerged as the top performer, achieving 96% accuracy and excelling particularly in harder-to-classify categories, such as Class 2. LSTM_LR followed closely with strong long-term pattern recognition, while RNN_LR performed adequately but lagged slightly in recall for minority classes. Confusion matrices, ROC curves, and F1-score charts all indicated that the hybrid GRU and LSTM models were top performers. The system also included real-time OLED visualization for live traffic feedback and worked entirely on the NodeMCU platform, proving both affordable and effective in edge settings.

That said, the system does have its limitations. For one, it relies heavily on RSSI and SNR for interpreting network behavior, both of which are sensitive to walls, interference, and physical movement. These factors could throw off detection accuracy in busier or less controlled environments. Plus, the models were trained in a fixed context, and while they performed well there, they may require retraining to remain effective in larger, more complex networks. While our system demonstrates reliable performance in controlled single-floor scenarios, its effectiveness in multi-floor, high-density access point (AP) environments would be considerably reduced. This limitation is primarily because of the restricted coverage and sensitivity of the onboard microstrip antenna of the NodeMCU. In such situation, signal attenuation through floors and the interference generated by overlapping APs would significantly hinder the ability of a single device to capture and classify deauthentication packets accurately. To address this, a distributed deployment strategy would be required, involving multiple NodeMCU units strategically positioned across floors to ensure adequate spatial coverage and reliable detection.

While the system does not capture packet contents, it still monitors device behavior, which raises ethical considerations. It is meant as a security tool for authorized admins, not a surveillance mechanism. Any use in the real world must be declared openly, with permission having been granted, as is obvious. Users must be informed, and measures are taken to limit misuse. In the future, improving its privacy features would help to make it beneficial without violating any ethical limits.

In summary, the integration of deep temporal modeling and lightweight classification within a compact embedded platform highlights the feasibility of deploying advanced security frameworks for wireless networks. Compared with state-of-the-art lightweight IDS systems for embedded platforms, which typically report detection accuracy in the 85–92% range, our system achieves improved performance (96%) while maintaining real-time inference and low-resource utilization. These results underline the novelty of combining temporal sequence modeling with logistic regression on an edge device and demonstrate that hybrid model architectures can provide both efficiency and enhanced detection accuracy for real-world IoT environments.

### Future work

With this promising result, subsequent research can overcome much of the most significant limitations found in development and deployment. One of the problems was the OLED display’s low memory, which caused bugs in attempting to include additional features. This restricted real-time visualization of key network metrics and excluded more complex indicators from integration. Moreover, NodeMCU’s lower RAM was a major constraint, as it was not possible to offload even a single parameter to third-party cloud services such as Firebase. This restricted the ability of the system to log data remotely or to perform off-device computations, which are critical for large-scale deployment and continuous monitoring. The other problem that was noticed was the weak reception of the signal, which impacted trilateration and RSSI-based attack detection adversely; it suggests the incorporation of signal amplification techniques or using external antennae.

Future work can expand on this work by integrating (RSSI) into trilateration-based localization frameworks. While RSSI in this paper was applied chiefly to signal quality evaluation and anomaly detection, its potential for precise spatial localization remains widely untapped. Trilateration, which computes a device’s position from distances to several access points, stands to drastically improve wireless localization precision when combined with RSSI fingerprinting. Since precise trilateration would necessitate several access points deployed across the environment, a single source would be insufficient. Combined in our system, trilateration could render the system far more powerful by enabling precise threat localization, increasing the granularity of anomaly detection, and improving overall system robustness to real-world deployments. Problems inherent to multipath fading, interference, and limited hardware capabilities of low-power devices, however, remain considerable obstacles. Incorporating trilateration in future research phases will not only provide more accurate positioning but also facilitate innovative real-time security monitoring and context-aware IoT services.

To further these horizons and allow the system to operate even more efficiently, the inclusion of state-of-the-art next-generation transformer-based models such as DeBERTa or ELECTRA would be an option. These would allow for greater contextual understanding and classification precision, especially with difficult or dynamic traffic conditions. Even more powerful edge-computing platforms such as the Raspberry Pi 4 Power Edition, coupled with state-of-the-art network analyzers, would also make better real-time signal processing and harvesting possible and further increase the accuracy of localization and detection.

It will also be necessary to scale up the diversity and size of the dataset to capture the strength and extensibility of the hybrid models across varying network settings and attack patterns. Probing model compression techniques (knowledge distillation, parameter sharing, and neural architecture search) will also facilitate efficient deployment on resource-limited devices without impairment. Use of ensemble methods’ predictions, which aggregate predictions from numerous transformer-based models, can support more robust classification certainty, especially for minority class labels. Finally, with domain-adaptive fine-tuning along with continuous learning paradigms, the detection system would be able to maintain high accuracy even in dynamic systems with changing network patterns over time.

## Data Availability

The datasets analyzed during the current study are available from the corresponding author on reasonable request.
